# Goblet Cell Carcinoids of the Appendix

**DOI:** 10.1155/2013/543696

**Published:** 2013-01-14

**Authors:** Nanna Holt, Henning Grønbæk

**Affiliations:** Department of Medicine V, Aarhus University Hospital, 44 Norrebrogade, 8000 Aarhus C, Denmark

## Abstract

Goblet cell carcinoid (GCC) tumors are a rare subgroup of neuroendocrine tumors almost exclusively originating in the appendix. The tumor most often presents in the fifth or sixth decade with a clinical picture of appendicitis or in advanced cases an abdominal mass associated with abdominal pain. Histologically tumors are most often positive for chromogranin A and synaptophysin, however, less homogenous than for classic appendix carcinoids. The malignant potential is higher than that for the classic appendix carcinoids due to local spread and distant metastases at diagnosis and the proliferation markers (Ki67 index) may determine prognosis. Octreotide receptor scintigraphy is usually negative while CT/MRI scans may be useful. Chromogranin A is usually negative and other biomarkers related to the mucinous component or the tumor (CEA, CA-19-9, and CA-125) may be used. Surgery is the main treatment with appendectomy and right hemicolectomy while patients with disseminated disease should be treated with chemotherapy. Overall 5-year survival is approximately 75%. The diagnosis and treatment of GCC tumorss should be restricted to high volume NET centers in order to accumulate knowledge and improve survival in GCC NET patients. The aim of this paper is to update on epidemiology, clinical presentation, and diagnostic markers including Ki67 index, treatment, and survival.

## 1. Introduction

Goblet cell carcinoids (GCCs) are, contrary to the normal carcinoids, mixed tumors with partly neuroendocrine differentiation and partly goblet cell type morphology [1–3], and GCCs are synonymous with adenocarcinoids, goblet cell tumors, and mucinous adenocarcinoids. They are assumed to arise from multipotential stem cells at the base of the crypts in the mucosa of the intestine. The cells often stain weakly positive for the neuroendocrine tumor markers chromogranin A and synaptophysin, at least in focal areas, and simultaneously produce mucin, like colorectal adenocarcinomas [4–7].

Goblet cell carcinoids were first described in 1974 as a separate group with the name goblet cell carcinoids by Subbuswamy et al. [8]. They described twelve tumors of the appendix, which showed histologic features different from both adenocarcinoma and the ordinary carcinoid of the appendix. They described the principal cell type as having a close resemblance to the normal goblet cell in the epithelium of the intestinal tract but found a considerable number of paneth and argentaffin cells in some of the tumors [8]. Before this, reports of similar cases were published in 1969 by Gagné et al. [9] where they described three tumors of the appendix but found common features, which they found unusual. Among other things, these common features were the association of nests, which were rich in enterochromaffin cells with mucus secreting glandular structures [9].

GCC almost exclusively occur in the appendix but may occasionally be found in other parts of the gastrointestinal tract [10, 11]. Watson and Alguacil-Garcia described back in 1987 eleven patients with mixed crypt cell carcinoma, which is the same as GCC. Six of them had primary tumor at the appendix, while five of them had tumors of the gastrointestinal tract. Of these five patients, one of them had a tumor located to the ileum, two of them had tumors located to the colon, and the last two patients had tumors located to the rectum [11]. Recently, Gui et al. reviewed the literature regarding extra-appendiceal locations of GCC and found that true primary extra-appendiceal GCC is extremely rare [10]. 

In the present paper we update on the recent status of GCC with focus on epidemiology, clinical presentation, diagnostic markers, treatment, and survival. 

## 2. Classification

According to Tang et al., GCC can be classified as typical GCC (*Group A*) and adenocarcinoma ex-GCC on the basis of the histologic features at the primary site of the tumor. The adenocarcinoma ex-GCC group can be further divided into signet ring cell type (*Group B*) and poorly differentiated adenocarcinoma type (*Group C*). The classification is based only on morphologic features at the primary site, that is, the appendix. *Group A* has well-defined goblet cells arranged in clusters or cohesive linear pattern, minimal cytologic atypia, minimal to no desmoplasia, minimal architectural distortion of the appendiceal wall, and degenerative change with extracellular mucin is acceptable. *Group B* has goblet cells or signet ring cells arranged in irregular large clusters, but lack of confluent sheets of cells, discohesive single file, or single cell infiltrating pattern, significant cytologic atypia, and desmoplasia and associated destruction of the appendiceal wall. *Group C* has at least focal evidence of goblet cell morphology, a component (>1 low power field or 1 mm^2^) not otherwise distinguishable from poorly differentiated adenocarcinoma, which may appear as either (a) gland forming, (b) confluent sheets or signet ring cells, or (c) undifferentiated carcinoma [12].

A recent study by Jiang et al. from 2012 shows a possible connection between GCC and schistosomiasis, which is the only potential risk factor for GCC identified to date [13]. They examined appendix samples from 3 patients with combined GCC and appendiceal schistosomiasis, 6 patients with GCC only, 12 patients with appendiceal schistosomiasis only, and 12 cases with normal appendix. In this study, their findings suggest that appendiceal schistosomiasis is associated with both increased proliferation and neuroendocrine differentiation of mucosal pluripotent crypt cells, and that it hereby may contribute to development of GCC [13]. However, these data needs to be confirmed by other research groups.

## 3. Epidemiology

Goblet cell carcinoids are considered a distinct entity of appendiceal tumors and occur in 0.3%–0.9% of appendectomies where they comprise 35%–58% of all appendiceal neoplasm [4, 14, 15], and less than 14% of all malignant tumors of the appendix [16]. Thus GCCs are extremely rare and in the period 1973–2001 with an incidence of approximately 0.05/100,000 per year compared to appendiceal endocrine tumors with an incidence of 0.63/1,000,000 per year in the SEER database.

They are most often seen in patients in their fifties or sixties with a second peak in the seventies [2, 16, 17], but McCusker et el., who have published the largest study regarding primary malignant neoplasms of the appendix, including GCC, described a large variation in age from 18 to 89 years [16]. In general this is 20–40 years later than the age peak for classical appendiceal neuroendocrine tumors.

They are more often seen in the Caucasian population than in any other group [16]. Some studies suggest a difference in age at time of diagnosis depending on whether the tumor is localized or disseminated, and with higher age at time of diagnosis when disseminated compared to localized disease [2].

There has been a disagreement whether GCCs have a preference regarding sex. Some studies found an increased frequency among women [12, 17], whereas others found no difference in prevalence between men and women [16, 18]. McCusker et al., who made a population-based study from the SEER database, 1973–1998, which is also the largest study to date with 227 GCC patients, found no difference in prevalence according to sex with 52% male and 48% female [16]. Therefore the overall impression is that the distribution among gender is equal in GCC patients. 

## 4. Clinical Presentation

Up to 60% of the patients present with acute appendicitis where the GCC is discovered by coincidence in connection with surgery for acute appendicitis [17, 19]. Hence, contrary to classical appendix carcinoids, which are much more common and are often found as discrete tumors at the apex of the appendix, adenocarcinoids often show a diffuse thickening involving the total length of the appendix or alternatively only the base of the appendix [20]. This may induce occlusion of the lumen of the appendix, which is the cause of appendicitis [21]. In cases with disseminated disease, the primary symptom is often abdominal pain associated with an abdominal mass and weight loss. However, only one study found a higher prevalence of abdominal pain combined with a palpable mass as primary symptom compared with symptoms of appendicitis [12]. In this study, by Tang et al., of 63 patients with GCC, most of the patients (63%) presented at an advanced clinical stage [12]. 

## 5. Diagnosis

The majority of patients will have surgery for acute appendicitis and the diagnosis is revealed after pathological examination of the inflamed appendix. Here most of the GCCs show scattered positivity for chromogranin A and synaptophysin [4–6, 12], and positivity for CK20 and CEA [4, 5]. This is in contrast to the classic appendix carcinoids where homogeneous staining for both chromogranin A and synaptophysin is most often seen. The proliferation index, Ki-67, which is of importance for the malignant potential in neuroendocrine tumors, has been studied thoroughly in a study by Tang et al. They categorized GCC patients in 3 groups according to histology; group A, B, and C, respectively, and showed that the average Ki-67 index increases within the groups, and that the survival rate is significantly reduced with increasing Ki67 index [12]. However, a recent study examined the role of Ki-67 in the prognosis of GCC [22]. Twelve GCC tumors were stained with MIB-1, a monoclonal antibody of Ki-67, to assess their cell proliferation and correlation with clinical and histologic parameters. In conclusion, they found that Ki-67 had no prognostic significance for GCC and therefore should not be used solely to determine treatment and surgical approach [22]. Another study with 26 GCC patients only showed a mean Ki67 index of 5 ± 3% (range 2%–13%) with significantly higher levels than typical appendix carcinoid tumors, but the study did not report on prognosis or survival [23]. A recent smaller study demonstrated higher Ki67 index in patients with disseminated disease (Ki67 > 10%–20%) compared to Ki67 index less than 5% in patients with localized disease [24]. Alsaad et al. investigated Ki-67 (MIB-1) immunostaining in 17 GCC patients and observed variation from 0% to 75% with index >2% in 41% of GCC tumors [25]. However, they did not find any correlation of Ki-67 with prognosis. We need larger studies with longer followup investigating the Ki67 proliferation marker as a prognostic marker in GCC patients.

The majority of GCC are localized in the appendix; however, in the case of disseminated disease, which is more prevalent in women, the ovaries and peritoneum (as carcinomatosis) are often involved. In contrast, metastasis to the lungs and liver are rare, compared to metastasis to these places from classic intestinal carcinoid tumors and adenocarcinomas [6, 11, 12, 18, 19, 26].

The prevalence of metastatic disease at presentation is high in GCC patients and ranges from 51% to 97%. Tang et al. observed disseminated disease in 63% of the GCC patients [12]. This is in contrast to a large study by McCusker et al., and other smaller studies, with disseminated disease in 17%–20% of the GCC patients [16, 18, 27]. However, most studies demonstrate that disseminated disease with metastasis is found more often in women than in men [17, 28, 29]. 

Chromogranin A is the most important biomarker for diagnosis of classic neuroendocrine tumors. However, in patients with GCCs chromogranin A is usually negative and no specific neuroendocrine markers have been observed. This may be related to the lack of endocrine secretory granules in the GCC cells and evident by only scattered or absent tumor staining for chromogranin A. In a small study, plasma chromogranin A and urinary 5-HIAA were assessed at the time of referral and during the follow-up period and CgA was elevated in only two of four patients with disseminated disease [24]. In patients with disseminated disease, epithelial markers and other markers related to the mucinous component or the tumor may be elevated. It is suggested to use CEA, CA-19-9, and CA-125 at presentation and during followup [30]. However, larger prognostic studies are warranted for the optimal use of biomarkers in GCC patients. 

The GCC tumor and metastases are difficult to visualize by imaging. As the presence of somatostatin receptors on goblet cells in general is sparse or lacking, somatostatin receptor scintigraphy (SRI) or Gallium-DOTANOC-PET scans are usually not useful [31]. A small study using octreotide receptor scintigraphy scan in 15 patients only showed minimal uptake in mesenteric lymph nodes in two of the four patients with metastases and were normal in the two other patients. Further, I-123 MIBG scans were normal in all patients and FDG-PET scans performed in two patients with metastatic disease by conventional imaging were negative in both patients [24]. FDG-PET may be useful in patients with increased metabolic activity and high ki67 index [32], but data in GCC patients are sparse, and there are no specific diagnostic studies of imaging focused on GCC [33]. Computer tomography (CT) scanning or magnetic resonance imaging (MRI) usually has a low sensitivity for local spread of the disease; however, these imaging techniques may be used to rule out metastasis to the lymphnodes and liver [33]. Lifelong screening for synchronous or metachronous malignancies is recommended. In addition, there might be an increased risk of secondary neoplasms [19, 33]. 

## 6. Treatment

Treatment of GCC is based on surgery, and because of its natural history and malignant nature, treatment recommendations are in general similar to intestinal adenocarcinomas. Localized stage I tumors may be treated with appendectomy alone. However, there has been disagreement whether simple appendectomy is sufficient to secure radicality, or whether the patients also need a right hemicolectomy [12, 17, 18, 21, 34]. In higher stages, a right hemicolectomy is recommended for nodal sampling, as GCC has shown increased risk for local lymph node metastases [12]. Two studies have shown beneficial effect of extensive surgery in infiltrative tumors provided there was no nodal involvement, and with no residual tumor during followup [18, 35]. However, another study showed similar 5-year survival rates for GCC patients with appendectomy alone and those who underwent right hemicolectomy. Interestingly, The SEER database have shown that only 42% of GCC patients receive right hemicolectomy [16, 19]. The European Neuroendocrine Tumor Society (ENETS) and the North American Neuroendocrine Tumor Society (NANETS) recommend a right hemi-colectomy [33, 36] as also advocated for in a recent study [12]. Likewise, some publications suggest a prophylactic removal of the ovaries in women due to the high incidence of metastases to the ovaries [12]. The age of the patient, menopausal status, and planned pregnancies have to be discussed with the patient [30]. Smaller studies have suggested that cytoreductive surgery with intraperitoneal chemotherapy (HIPEC) may be an option in GCC patients with peritoneal carcinomatosis [29, 37]. In a study from 2004, the overall median survival was 18*·*5 months (range 3–95) in 22 GCC patients treated with cytoreductive surgery and HIPEC [29]. A recent Swedish study showed even better long-term survival with a median survival of 30 months (range 9–38 months) and a 1 year survival rate of 80% and 20% after 3 year [38]. 

After surgery, followup with blood samples and imaging (CT or MRI) is recommended while the role of PET scans has not yet been clarified. In contrast to classic appendix neuroendocrine tumors, CgA determination is not recommended. Instead CEA, CA-125, and CA-19-9 are suggested as tumor markers [33], please see above. 

When disseminated at time of diagnosis, debulking surgery is recommended when possible [14] followed by adjuvant chemotherapy with regimens similar to colorectal adenocarcinoma. There are case reports of regimens using streptozotocin and 5FU or platin-based therapies in combination with etoposide, while even more aggressive combinations using FOLFOX/FOLFIRI (5-fluorouracil/leucovorin combined with irinotecan and oxaliplatin) may also be used. Evident guidelines for choice of chemotherapy are still lacking [17–19, 27]. The ENETS Guidelines 2012 advocate for the use of 5-fluorouracil-based combination regimen as first line therapy [33]. However, we need more and larger studies on chemotherapy regimens. 

## 7. Prognosis

Subbuswamy et al., who gave this tumor type its name GCC in 1974, thought that the histology and prognosis suggested a tumor of very low grade of malignancy, comparable with the behavior of argentaffin and nonargentaffin carcinoid of the appendix [8]. Later on, other studies have found a worse prognosis. The malignant potential of GCC is higher than for the classic appendix carcinoids, and some of them even have a course of the disease similar to gastrointestinal adenocarcinomas [16, 17]. However, the precise histogenesis and cause of the malignant progress are still unknown [2, 14, 29].

The majority of patients survive for many years, and in a large study by McCusker et al. GCCs are associated with an 80% 5-year survival rate and 65% 10-year survival rate, respectively [16]. In this study, 227 patients with GCC were included, and they found that the overall biologic behavior of GCC in their series was intermediate between that of adenocarcinomas and carcinoid tumors in term of age at diagnosis, extent of disease spread at diagnosis, and number of cases with lymph node involvement [16]. This, acceptable survival rate can be explained by the fact that most of the GCC are still localized at time of diagnosis. In contrast to this, Pham et al. only observed a 45% 5-year survival rate [17]. This study included 57 patients with GCC, and they found a tendency for GCC to occur more frequently in women and simultaneously that half of the female patients had metastasis to the ovaries at time of initial presentation [17]. Tang et al., who made a study with 63 patients, separated the tumors into 3 groups according to histology and found a 5-year survival rate that decreased from 100% to 0%, from group A to group C, respectively [12]. Therefore, they concluded that careful evaluation of the morphologic features of GCC and appropriate pathologic classification are crucial for clinical management and prediction of outcome [12]. This seems to illustrate that when GCCs are discovered at an early stage with a low Ki67 index, they have an outcome similar to the classic appendiceal neuroendocrine tumors, while patients with high Ki67 index and disseminated disease at time of diagnosis have an outcome similar to gastrointestinal adenocarcinomas [12, 33]. In patients with cytoreductive surgery and HIPEC, early referral for these treatment regimens seems to improve prognosis [38].

## 8. Conclusion 

GCC is a rare neuroendocrine tumor type and should be diagnosed and treated in high volume NET centers capable of offering the multidisciplinary approach as recommended by NET societies. This may hopefully lead to international multicenter clinical trials, increase the knowledge of GCC, and hopefully improve survival for GCC patients in the future (Table 1).

## Figures and Tables

**Table 1:** Suggested algorithm for the treatment and followup from the literature cited in the paper.

	Histology:
	(i) Scattered staining for chromogranin A and synaptophysin, along with mucus.
	(ii) Ki67 index.
	(iii) Staging according to Tang et al. [12].
	Imaging:
Diagnostic procedures	(i) CT/MRI of the chest and the abdomen/pelvis
	(ii) MRI of the abdomen/pelvis
	(iii) Somatostatin receptor scintigraphy, Ga^68^-PET scans are usually negative
	(iv) FDG-PET and MIBG-PET scannings are usually negative
	Biochemistry:
	(i) CEA, CA-19-9, CA-125
	(ii) Chromogranin A and U-5HIAA usually normal

	Appendectomy
Surgical therapy	Hemicolectomy (standard surgical treatment)
	Debulking surgery when possible

Medical therapy	5-fluorouracil-based chemotherapeutic regimen
	Cytoreductive surgery combined with HIPEC in selected cases

	(i) Clinical: abdominal pain, weight loss
Followup	(ii) Biochemistry: CEA, CA-19-9, and CA-125
	(iii) Imaging: CT or MRI every 3–6 months, then yearly, mimicking the guidelines for colorectal adenocarcinoma. Lifelong followup.
